# Production of the *Escherichia coli* Common Pilus by Uropathogenic *E. coli* Is Associated with Adherence to HeLa and HTB-4 Cells and Invasion of Mouse Bladder Urothelium

**DOI:** 10.1371/journal.pone.0101200

**Published:** 2014-07-18

**Authors:** Zeus Saldaña, Miguel A. De la Cruz, Erika Margarita Carrillo-Casas, Laura Durán, Yushan Zhang, Rigoberto Hernández-Castro, José L. Puente, Yehia Daaka, Jorge A. Girón

**Affiliations:** 1 Department of Molecular Genetics and Microbiology, Emerging Pathogens Institute, University of Florida, Gainesville, Florida, United States of America; 2 Department of Anatomy and Cell Biology, University of Florida College of Medicine, Gainesville, Florida, United States of America; 3 Departamento de Ecología de Agentes Patógenos, Hospital General Dr. Manuel Gea González, Tlalpan, Mexico City, México; 4 Departamento de Microbiología Molecular, Instituto de Biotecnología, Universidad Nacional Autónoma de México, Cuernavaca, Morelos, México; IRCSS - Istituto di Ricerche Farmacologiche Mario Negri, Italy

## Abstract

Uropathogenic *Escherichia coli* (UPEC) strains cause urinary tract infections and employ type 1 and P pili in colonization of the bladder and kidney, respectively. Most intestinal and extra-intestinal *E. coli* strains produce a pilus called *E. coli* common pilus (ECP) involved in cell adherence and biofilm formation. However, the contribution of ECP to the interaction of UPEC with uroepithelial cells remains to be elucidated. Here, we report that prototypic UPEC strains CFT073 and F11 mutated in the major pilin structural gene *ecpA* are significantly deficient in adherence to cultured HeLa (cervix) and HTB-4 (bladder) epithelial cells *in vitro* as compared to their parental strains. Complementation of the *ecpA* mutant restored adherence to wild-type levels. UPEC strains produce ECP upon growth in Luria-Bertani broth or DMEM tissue culture medium preferentially at 26°C, during incubation with cultured epithelial cells *in vitro* at 37°C, and upon colonization of mouse bladder urothelium *ex vivo*. ECP was demonstrated on and inside exfoliated bladder epithelial cells present in the urine of urinary tract infection patients. The ability of the CFT073 *ecpA* mutant to invade the mouse tissue was significantly reduced. The presence of ECP correlated with the architecture of the biofilms produced by UPEC strains on inert surfaces. These data suggest that ECP can potentially be produced in the bladder environment and contribute to the adhesive and invasive capabilities of UPEC during its interaction with the host bladder. We propose that along with other known adhesins, ECP plays a synergistic role in the multi-step infection of the urinary tract.

## Introduction

Bacterial adherence is the first step in host tissue colonization and development of disease and it is generally a multi-factorial event that involves the participation of fimbrial and non-fimbrial adhesins orchestrated, in turn, by a myriad of regulatory elements influenced by environmental and host signals at different stages during the infectious process [1]. Urinary tract infections represent a significant public health problem, particularly in women, and an economic burden due to treatment cost [2]. In the urinary tract, *Escherichia coli* strains of fecal origin are able to ascend the urethra and colonize the bladder through recognition of uroplakin, a mannose-rich proteinaceous plaque found at the luminal surface of bladder epithelial cells. Pili-mediated bacterial attachment triggers a myriad of host-initiated processes including cytokine production, apoptosis and exfoliation [3], [4]. Binding and invasion of uropathogenic *E. coli* (UPEC) into bladder epithelial cells is mediated by the FimH adhesin of the type 1 pilus (T1P). UPEC invasion involves fusiform vesicles, cyclic AMP, Toll-like receptor 4 (TLR4) and integrins [5], [6], [7]. A recent study showed that T1P-mediated bacterial invasion of bladder epithelial cells is regulated by dynamin 2, a guanosine triphosphatase, and its partner endothelial nitric oxide synthase [8]. During cell invasion, UPEC is either released back into the urine or into the cytoplasm. Once inside the cytoplasmic space the bacteria employs T1P and the Ag43 protein to form an organized intracellular bacterial community (IBC) resembling a biofilm structure thus evading host immune defenses [9]. Presumably, these IBCs represent a quiescent intracellular reservoir and a source of recurrent infections [11]. Whereas previously seen only in mouse bladders and urine, these stages have been identified in human urine samples and human biopsies [12], demonstrating clearly that these events do occur as part of the human condition.

Several pili types, including T1P, pyelonephritis-associated pilus (Pap) and S pili, and the Dr adhesins of UPEC contribute to infections in the urinary tract [4]. However, the genomes of prototypic UPEC strains CFT073 and UTI89 contain several putative pili operons, but it is unclear which of these operons are expressed or functional in the host.

Clinical meningitis-producing *E. coli* (MENEC) strains produce a fimbrial structure called “meningitis-associated temperature-dependent fimbriae” or Mat upon growth in Luria-Bertani broth at 26°C [13], [14]. It was originally thought that the Mat fimbriae was not produced by intestinal pathogenic *E. coli*; however, it was later shown that the production of Mat fimbriae was not restricted to MENEC strains or to growth at temperatures below 37°C [15]. We found Mat fimbriae in 75% of a collection of intestinal and extra-intestinal *E. coli* strains and that the structural pilin gene *matB* is widely distributed and highly conserved amongst *E. coli* strains. For these reasons we proposed to call these fimbriae “*E. coli* common pilus” or ECP [15]. The presence of ECP enhances the adhesive properties of normal flora (NFEC), enterohemorrhagic (EHEC), enteroaggregative (EAEC), enteropathogenic (typical and atypical EPEC strains) and enterotoxigenic *E. coli* (ETEC) to cultured epithelial cells, suggesting that ECP may function as intestinal adherence factor for these *E. coli* [15], [16], [17], [18]. Expression of the ECP operon is under positive transcriptional regulation of EcpR (called MatA in MENEC), an activator protein that also regulates its own transcription [19]. MatA also regulates flagella by an unknown mechanism [20]. ECP plays a key role in bacteria-to-bacteria interactions within biofilms [21]. A homolog of the *ecp* operon exists in *Klebsiella pneumoniae*, an organism also capable of causing UTIs, and nearly 100% of clinical strains tested produced ECP, in association with the MR/K fimbriae, when adhering to cultured human epithelial cells [22]–[23]. Given the wide distribution and role of ECP among the different pathotypes of *E. coli* and *Klebsiella*, the aim of this study was to investigate the contribution of ECP to the adherence and invasion capabilities of UPEC.

## Materials and Methods

### Ethics Statement

Animal care use was reviewed and approved by the University of Florida IACUC.

### Bacterial strains and culture conditions

Bacterial strains and plasmids used in this study are listed in Table 1. Bacterial cultures were routinely obtained in Luria-Bertani (LB) broth with or without antibiotics [100 µg/ml (ampicillin) or 50 µg/ml (kanamycin)] after overnight growth with shaking at 37°C. To determine optimal conditions for ECP induction, CFT073 was grown overnight in LB and Dulbecco's Modified Eagle Medium (DMEM) (Invitrogen) at 26°C and 37°C with shaking [15].

**Table 1:** Bacterial strains and plasmids used in this study.

Strains	Notes	Reference
CFT073	Clinical isolate	[35]
CFT073Δ*ecpA*	Wild-type strain mutated in *ecpA*::km	This study
CFT073Δ*ecpA* (pMAT9)	Δ*ecpA* complemented with pMAT9	This study
F11	Clinical isolate	[36]
F11Δ*ecpA*	Wild-type strain mutated in *ecpA*::km	This study
F11Δ*ecpA*(pMAT9)	Δ*ecpA* complemented with pMAT9	This study
UTI89	Clinical isolate	[37]
Plasmids		
pKD46	Red recombinase system plasmid	[27]
pKD4	Kanamycin cassette	[27]
pMAT9	*ecpAB* (*matBC*) in pSE380	[14]

### Antibodies

For the various immunoassays performed, we used highly absorbed rabbit antibodies specific for ECP, which were available from previous studies [15], [24], [25]. Anti-DnaK was used in Western blots to ensure that equal amounts of proteins were loaded onto the gels.

### Cell line cultures

Human HeLa (cervix) and HTB-4 (bladder) (also known as T24) epithelial cells were maintained in DMEM with conventional antibiotics and 5% fetal bovine serum at 37°C in an incubator with 5% CO_2_ atmosphere. For adherence and invasion assays, cell monolayers were propagated in the appropriate culture medium until 80% confluent, washed with phosphate buffer saline (PBS, pH 7.4) and transferred to 24-well plates containing DMEM with 0.5% D-mannose and with or without glass coverslips [15]–[18].

### Interaction with cultured eukaryotic cells

HeLa and HTB-4 cells were infected with 10 µL (∼10^7^ bacteria) of a DMEM overnight culture of the indicated strains. To avoid cytotoxicity and cell lysis observed after prolonged incubation periods of cultured epithelial cells with UPEC strains, adherence assays were carried out for 2 h as previously described [15]–[18]. After the 2 h incubation period, the monolayers were washed twice with PBS to remove unbound bacteria. The cells were then fixed with 2% formaldehyde in PBS for Giemsa staining and immunofluorescence microscopy (IFM) as previously described [15]. Primary anti-ECP antibodies were added for 1 h in 10% horse serum in PBS followed by the appropriate secondary species-specific Alexa-Fluor 488 conjugates and then visualized using an Axio Imager1.0 Zeiss microscope (Zeiss). For comparative analysis of the invasive capabilities of the wild-type strain and the mutant, the gentamycin-protection assay was employed [26]. Briefly, after the 2 h incubation, the wells were washed as before and replenished with 1 mL of DMEM containing gentamycin (50 µg/mL) to kill extracellular bacteria and incubated for additional 2 h. For quantification of adherence and invasion, the cells were lysed with a solution of 0.5% Triton X-100 and after homogenization 10-fold serial dilutions were plated onto LB agar plates with the appropriate antibiotics to determine total colony-forming units (CFU). The number of adherent bacteria was calculated by subtracting the number of invasive bacteria in the assay with gentamycin from the number of total bacteria obtained in the assay without gentamycin. The results shown are the mean of 3 experiments performed in triplicate on different days. Statistical analysis was done using the Student's t-test.

### Ex-vivo infection of mice bladder

Even size sections (2 mm × 2 mm) of murine bladder transitional epithelium (urothelium) were infected in triplicate *in vitro* for 2 h at 37°C with UPEC strains. Animal care use was approved by the UF IACUC. After infection, the bladder tissues were washed 3 times with PBS and fixed in 4% paraformaldehyde in PBS for 16–24 h and stored at 4°C before thin sectioning and IFM. After washing with PBS each wash for 20–30 min the tissues were transferred to a solution of 20% sucrose in PBS for 1–2 days at 4°C. The tissues were then processed for sectioning and reacted with anti-ECP antibodies (1∶1,000) and goat anti-rabbit IgG conjugated to Alexa-Fluor 488 (1∶100) (Molecular Probes). Cellular and bacterial DNA was stained briefly with Hoechst nucleic acid stain (Molecular Probes), washed with PBS, mounted on glass slides, and then viewed in an Axio Imager1.0 Zeiss microscope (Zeiss). Replica samples were washed and viable bacteria, in the absence or presence of gentamycin, were enumerated by plating ten-fold serial dilutions onto LB agar containing the appropriate antibiotic. Statistical significance of these results was determined using the unpaired parametric Student's t-test.

### Construction of UPEC non-polar *ecpA* mutants

UPEC strains F11 and CFT073 were targeted for mutagenesis of *ecpA* following the procedure reported by Datsenko and Wanner [27]. Briefly, we used specific primers G60 and G61 (Table 2) to generate a PCR fragment containing *ecpA* sequences flanking a kanamycin cassette, using DNA of plasmids pKD4 as template. This fragment was electroporated into competent UPEC strains carrying plasmid pKD46 encoding the lambda Red recombinase, which expression was induced by adding L-(+)-arabinose (Sigma) at a final concentration of 10 mM. The mutation was confirmed by PCR using primers flanking *ecpA* (G90 and G91), as well as primers inside the kanamycin cassette (K1 and K2), and chromosomal DNA from Km resistant Ap sensitive colonies as template. The loss of ECP in the mutants was assessed by several immunoassays using antibodies against ECP. The *ecpA* mutants were complemented with *ecpAB* on plasmid pMAT9 to restore ECP production [14]. The *ecp* genes from plasmid pMAT9 were induced with 0.1 mM isopropyl-β-D-thiogalactopyranoside (IPTG) (Promega).

**Table 2:** Primers used in this study.

Name	Sequence	Use
G60	GTTCTGGCAATAGCTCTGGTAACGGTGTTTACCGGCGTGTAGGCTGGAGCTGCTTC	Mutagenesis
G61	TTAACTGGTCCAGGTCGCGTCGAACTGTACGCTAACCATATGAATATCCTCCTTAG	Mutagenesis
G90	AACAGCAATATTAGGGGCGTG	Screening mutants
G91	GGATAACAGCAGAGCGAGAAG	Screening mutants
K1	GCCCAGTCATAGCCGAATAGCCT	Screening mutants
K2	CGGTGCCCTGAATGAACTGCAGG	Screening mutants

### Analysis of EcpA production by SDS-PAGE and immunoblotting

Overnight bacterial cultures obtained from DMEM were adjusted to an absorbance of 1.1 at OD_600_. Equal numbers of bacteria were used to prepare whole-cell extracts after treatment with acidified water (pH 1.2), boiling for 5 min, addition of SDS-PAGE sample buffer and neutralization with 1N NaOH, as previously described [28]. The samples were electrophoresed in 16% polyacrylamide gels under denaturing SDS-PAGE conditions [29]. Detection of DnaK with anti-DnaK monoclonal antibody (MBL International) served as a control for equal amounts of protein loaded onto the gels. The proteins were electroblotted onto PVDF membranes, blocked with 1% dry milk, and the immobilized proteins were reacted with primary antibodies against ECP, followed by incubation with goat anti-rabbit IgG conjugated to peroxidase (Sigma Aldrich). Reactive bands were visualized using a chemo-luminescent reagent (Amersham).

### Ultrastructural analysis of ECP production by electron microscopy

Bacterial cultures were spotted onto 300-mesh carbon-Formvar copper grids, negatively stained with 10 µL of 1% phosphotungstic acid (pH 7.4) for 5 min, and analyzed for the presence of pili by transmission electron microscopy (TEM). Immuno-electron microscopy (IEM) studies were performed to confirm the presence of ECP by incubating the bacteria for 1 h with rabbit anti-ECP antibody (diluted 1∶10) in PBS containing 10% BSA and 1 h incubation with goat anti-rabbit IgG conjugated to 10-nm gold particles diluted 1∶10 (BB International), as previously described [25].

### Biofilm formation assay

*E. coli* strains were tested for biofilm formation onto glass coverslips in 24-well plates (Cellstar). Fifty μL of bacteria were added to each well containing 1 ml of DMEM supplemented with 0.5% D-mannose and incubated for 24 h at 37°C. The biofilms were fixed with 2% formaldehyde, stained with a solution of Giemsa and visualized under light microscope. For quantitative determinations, absorption of Crystal Violet dye by biofilms was measured at 595 nm with methanol as previously described [15].

### Motility assay

CFT073 and derivative strains were spiked on 0.3 % motility LB agar. After overnight incubation at 37°C, the halos obtained represented bacteria capable of swimming. Except for the Δ*fliC* negative control, all other strains showed motility.

### Presence of *ecpA* among UTI isolates

*E. coli* isolates collected from January 2010 to December 2011 were obtained from midstream urine samples of women clinically diagnosed with acute UTI from the Urology Service at the General Hospital "Dr. Manuel Gea González" in Mexico City. Bacteria included in this work were from cultures with counts >10^5^ CFU/mL or more. Isolates were identified by MicroScan 4 walkaway (Dade Behring). Pure cultures were maintained at −70°C in Brain-Heart infusion broth/glycerol 50%. Genomic DNA was isolated using a DNeasy blood and tissue kit (Qiagen) according to manufacturer's instructions. PCR amplifications were performed using a TopTaq master mix kit (Qiagen). For *ecpA* amplification, a set of primers (5′-TGAAAAAAAAGGTTCTGGCAATAGC-3′) and (5′-CGCTGATGAGGAGAAAGTGAA-3′) were used for the initial PCR. A second set of primers (5′-GTGACATGGCAAAATGATTACAGC-3′) and (5′-TCACGGGAATGAACTTATCACCC-3′), were used for *ecpRB* amplification to search for a possible modified form of *ecpA* in PCR-negative strains. UPEC strain CFT073 was used as a positive control.

### Analysis of urine sediments from UTI patients

Three mL of midstream urine from de-identified patients with acute UTI were centrifuged at 3000 rpm for 30 min at 4°C and the cell pellet was washed twice with PBS, placed in glass coverslips, and fixed with 3% paraformaldehyde for 15 min. An urine sample from a clinically healthy woman was used as a negative control. The preparations were PBS-washed two times and blocked with PBS-10% horse serum for 20 min at room temperature. A 1∶1500 dilution of the primary anti-EcpA antibody was added to the samples and incubated overnight at 4°C. After washing, secondary anti-rabbit IgG antibodies (Alexa-Fluor 488, green) were incubated for 40 min. The epithelial cells were stained for 1 h at room temperature with phalloidin-rhodamine diluted 1∶120 and washed two times with PBS. The preparations were counterstained with VectaShield (Vector Laboratories) that contains DAPI, and mounted on glass slides. Microscopical analysis was done with a Leica DM100 immunofluorescence microscope (Leica Microsystems) and microphotographs were taken with an Olympus FV100 confocal microscope (Olympus America Corporate).

## Results

### PCR-based survey of *ecpA* among UTI *E. coli* isolates

A total of 172 *E. coli* strains isolated from women with acute UTI with ages ranging between 12–44 years (mean age of 33.9 years) attending the General Hospital Dr. Manuel Gea González in Mexico City, were analyzed for the presence of *ecpA* by PCR. One hundred and seventy (98.83%) isolates were positive for *ecpA* and the 2 *ecpA*-negative strains were also negative for *ecpR* and *ecpB* genes flanking *ecpA*, indicating that some strains possess the *ecpA* gene with genetic variations or have lost *ecpA*. These data show that the majority of UPEC strains contain the *ecpA* gene.

### Demonstration of ECP production on prototypic UPEC strains

**Figure 1:**
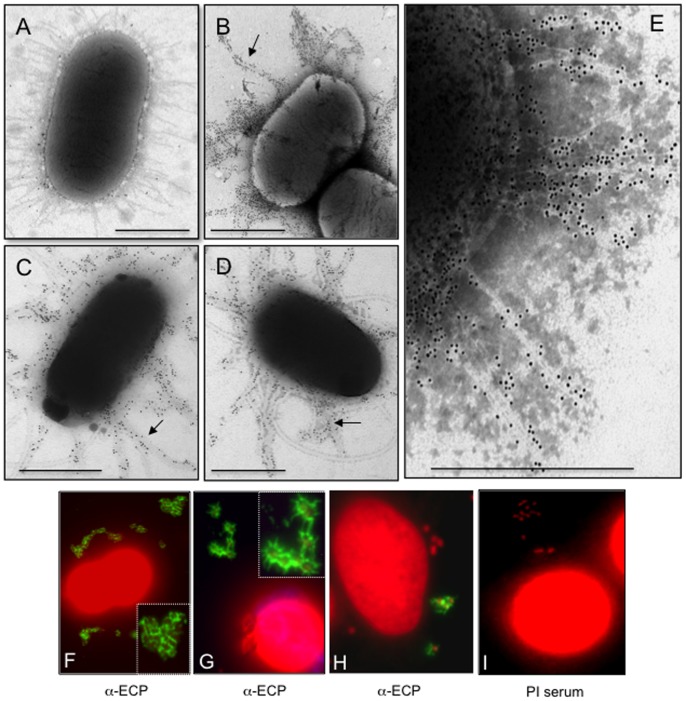
Demonstration of ECP on UPEC strains. (A) CFT073 displays peritrichous pili after growth in LB at 26°C with shaking and after incubation with pre-immune serum followed by negative staining and TEM observation. (B–D) Immunogold labeling of ECP (indicated by arrows) produced by F11 (B), CFT073 (C), and UTI89 (D) strains grown in LB at 26°C with anti-ECP antibodies and rabbit anti-IgG gold conjugate. (E) Magnification of labeled ECP on CFT073. Scale bars represent 500 nm. (F–H) Detection of ECP (green) on UPEC strains [F11 (F), CFT073 (G), UTI89 (H)] after 2 h of incubation with HeLa cells at 37°C and reacted with anti-ECP antibodies and Alexa-Fluor 488-conjugated secondary antibody. Cellular and bacterial DNA was stained with propidium iodide (red). Panel I is the negative control CFT073 incubated with preimmune serum. Images were taken with a 60× objective. The insets in panels F and G are high magnifications of the areas within the corresponding panels and show a fibrillar network (green) between bacteria formed by ECP.

We tested three prototypic UPEC strains F11, UTI89, and CFT073 for production of ECP after growth in LB at 26°C by TEM and IEM. These strains displayed pili seen as thin (3-nm wide) flexible fibers that protruded from the periphery of the bacterial surface when observed at high magnifications (*e. g*. >30,000X) under the electron microscope (Fig. 1A). These pili are morphologically distinct from the well-characterized rigid type 1 pili (T1P), which are thicker (7 nm) in diameter and are also produced by UPEC strains [30]. The identity of ECP on these 3 strains was confirmed by IEM using specific anti-ECP antibodies and species-specific IgG 10-nm gold conjugate (Fig. 1B–E). The anti-ECP antibodies raised against EHEC O157:H7 ECP do not cross react with T1P [15]. No reactivity was seen on the bacteria or the pili with rabbit pre-immune serum (Fig. 1A).

Next, in order to determine the biological relevance of the expression of ECP on UPEC adherence, we sought to investigate whether ECP was produced by UPEC strains adhered to cultured HeLa cells. The IFM images shown in Figure 1F-H are compelling evidence that ECP are distributed around the adhering bacteria and suggest that they promote bacteria-bacteria interactions. No reactivity was seen in the negative control containing preimmune serum, confirming the specificity of the antibodies and the reaction (Fig. 1I). These data clearly indicate that UPEC strains are capable of producing ECP under conditions that might mimic a host environment, namely the presence of epithelial cells, low oxygen tension, and body temperature (37°C).

**Figure 2:**
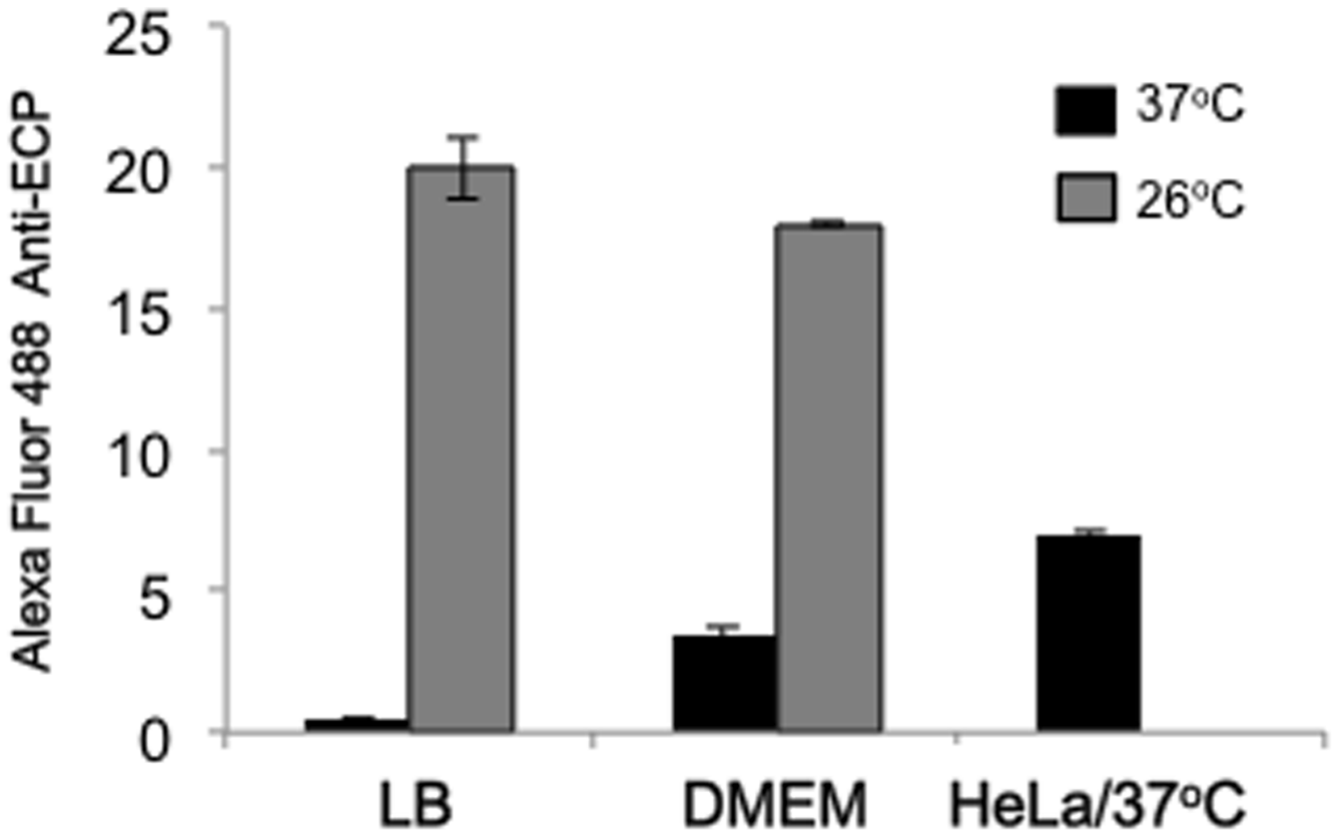
Optimal conditions for induction of ECP. Production of ECP in CFT073 bacteria grown in LB or DMEM at 26°C or 37°C was measured by flow cytometry using primary anti-EcpA antibodies and Alexa-Fluor 488 conjugate. In addition, levels of ECP production were determined in the presence of HeLa cells at 37°C. Standard deviations are represented by error bars and represent the average of all the results obtained from the 3 experiments performed.

In addition, we compared levels of ECP production after growth of CFT073 in LB and DMEM at 26°C and 37°C by flow cytometry. For comparison, we also measured ECP production on bacteria recovered from the supernatants of infections of HeLa cells. The data show that ECP is best produced at 26°C than at 37°C in LB followed by DMEM and that the bacteria produce ECP in the presence of HeLa cells at 37°C (Fig. 2).

### Analysis of *ecpA* null mutants

**Figure 3:**
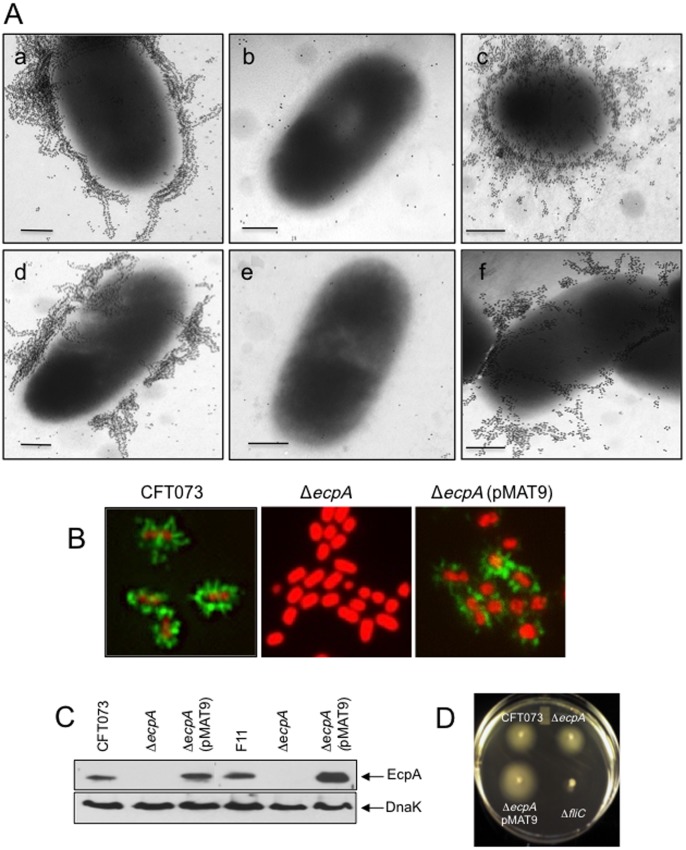
Analysis of derivative *ecpA* mutants. (A) Immunogold labeling of ECP produced by (a) CFT073, (b) CFT073 Δ*ecpA*, (c) CFT073 Δ*ecpA* (pMAT9), (d) F11, (e) F11 Δ*ecpA*, and (f) F11 Δ*ecpA* (pMAT9) with anti-ECP antibodies and rabbit anti-IgG gold conjugate. Scale bars represent 500 nm. (B) Immunofluorescence of UPEC strains with anti-ECP antibodies and rabbit anti-IgG and Alexa-Fluor 488-conjugated secondary antibody (green). Cellular and bacterial DNA was stained with propidium iodide (red). Images were taken with a 100× objective and magnified 5X. (C) Immunoblot showing EcpA production in CFT073, CFT073 Δ*ecpA* (pMAT9), F11 and F11 Δ*ecpA* (pMAT9), but not in the CFT073 Δ*ecpA* or F11 Δ*ecpA* mutants. DnaK detection was used as loading control of equal amounts of bacteria onto the gels. (D) Motility of UPEC strains in motility agar. Except for the CFT073 Δ*fliC* all other strains showed motility.

To determine the role of ECP in the interplay of UPEC with host epithelial cells we deleted the *ecpA* gene from CFT073 and F11 UPEC strains. The resulting isogenic ECP mutants did not show pili reacting with the anti-ECP antibodies, as determined by gold labeling IEM (Fig. 3A). To restore ECP production, the *ecpA* mutants were transformed with plasmid pMAT9, which harbors the *ecpA* (pilin gene) and *ecpB* (chaperone pilus biogenesis gene) [14]. Using IFM and anti-ECP antibodies a distinct pattern of peritrichous fluorescent fibers associated with the wild-type bacteria and the complemented strain was observed, while no fluorescent pili were seen on the mutants (Fig. 3B). Further, treatment of bacterial whole cell extracts with HCl to dissociate the pili into pilin subunits identified the EcpA pilin in wild-type and complemented strains, but not in the *ecpA* mutants in immunoblots (Fig. 3C). We also found that the CFT073 *ecpA* mutant was still motile, indicating that the lack of ECP does not interfere with motility (Fig. 3D).

### Isogenic *ecpA* mutants are hampered in their ability to adhere to HeLa and HTB-4 cells

**Figure 4:**
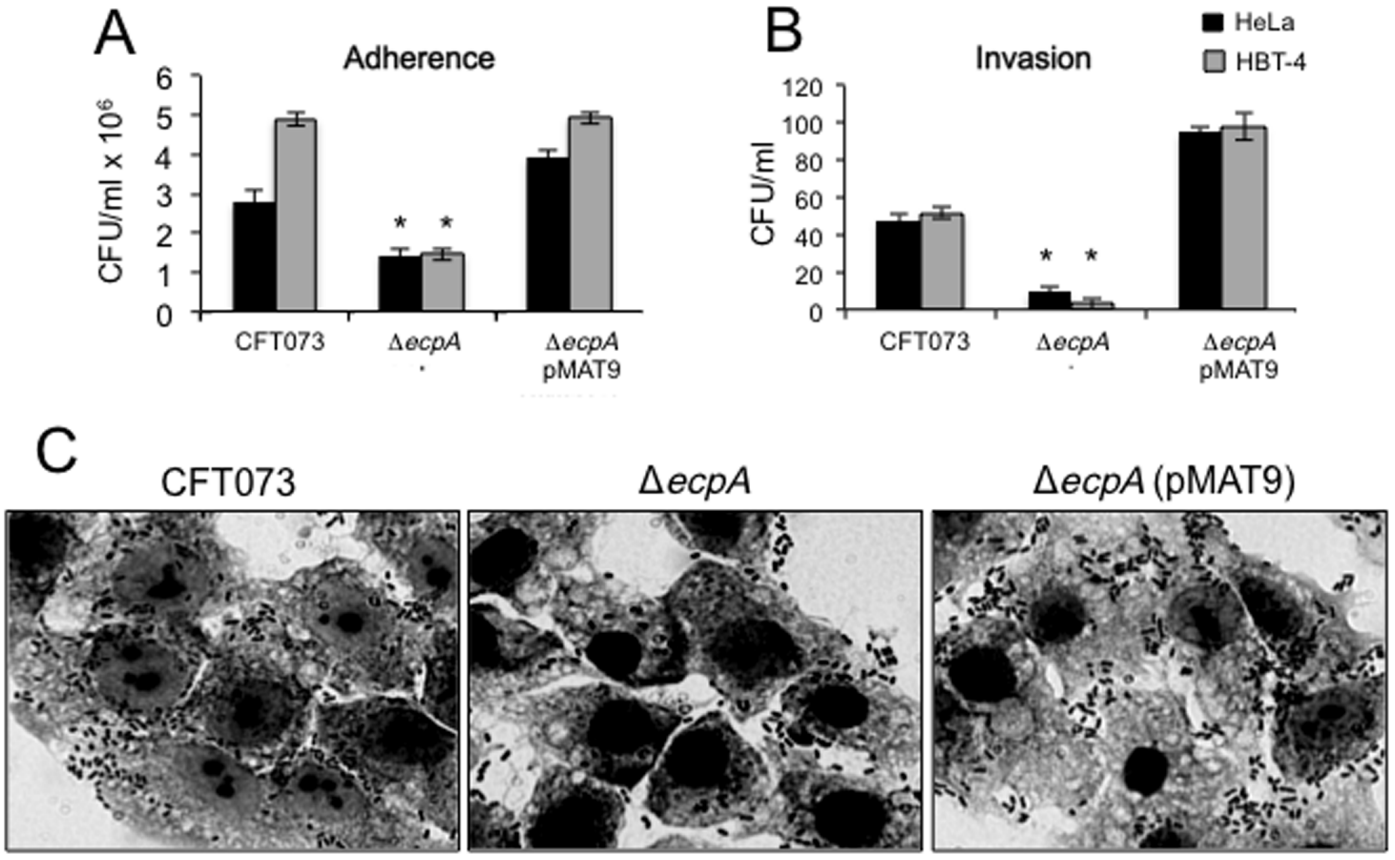
Role of ECP in adherence of UPEC to HeLa and HTB-4 cells. (A) Comparison of adherence levels of wild-type strain CFT073 and isogenic *ecpA* mutant to HeLa and HTB-4 cells at 37°C, as determined by plating out serial dilutions of adhering bacteria. (B) Comparison of invasion capabilities of HeLa and HTB-4 cells by the indicated UPEC strains as determined by the gentamycin-protection assay. For panels A and B standard deviations are represented by error bars and represent the average of all the results obtained from the 3 experiments performed. *, Statistically significant with respect to the wild-type strain (*p*<0.05). (C) Giemsa staining of HeLa cell monolayers infected for 2 h with UPEC CFT073 strains showing reduction of adherence in the isogenic *ecpA* mutant. Images were taken with a 60× objective.

At this point the data suggested that ECP acts as an adhesive factor that could contribute to the overall adherence properties of UPEC. To further judge the biological significance of ECP in bacterial adherence, the ability of the CFT073 and F11 *ecpA* mutants to adhere to HeLa and bladder HTB-4 cells was compared with the parental strains. A significant reduction (p<0.05) in adherence was observed for both mutants to both cell lines. CFT073 Δ*ecpA* manifested 52% reduction in adherence to HeLa cells and was 3 times less adherent to HTB-4 bladder epithelial cells than the wild-type strain (Fig. 4A). The reduced adherence of the CFT073 *ecpA* mutant was confirmed by light microscopy observation (Fig. 4C). The F11 Δ*ecpA* was reduced by 57% in adherence to HeLa cells (data not shown). The residual adherence in the mutants is not surprising considering that these bacteria possess numerous adhesive systems. Complementation of the *ecpA* mutants with *ecpAB* in plasmid pMAT9 restored adherence to wild-type levels in both strains (Fig. 4A and C).

### Role of ECP in cell invasion

It has been reported before that some UPEC strains are able to invade host bladder cells and to reproduce inside the host cell forming IBCs and that T1P contribute directly to the process of internalization [5]–[8]. Thus, we assayed CFT073 Δ*ecpA* for invasion of HeLa and HTB-4 cells through the gentamycin-protection assay. The data shown in Fig. 4B clearly indicate that the *ecpA* mutant was significantly reduced in invasion (*p*<0.05) to these cell lines suggesting that ECP presumably also plays a role in the invasive properties of this *E. coli*. Restoration of ECP production in the mutant carrying plasmid pMAT9 recovered the invasive properties of this strain (Fig. 4B). This is to our knowledge the first compelling evidence that ECP participates both in adherence and cell invasion.

### Role of T1P in adherence

**Figure 5:**
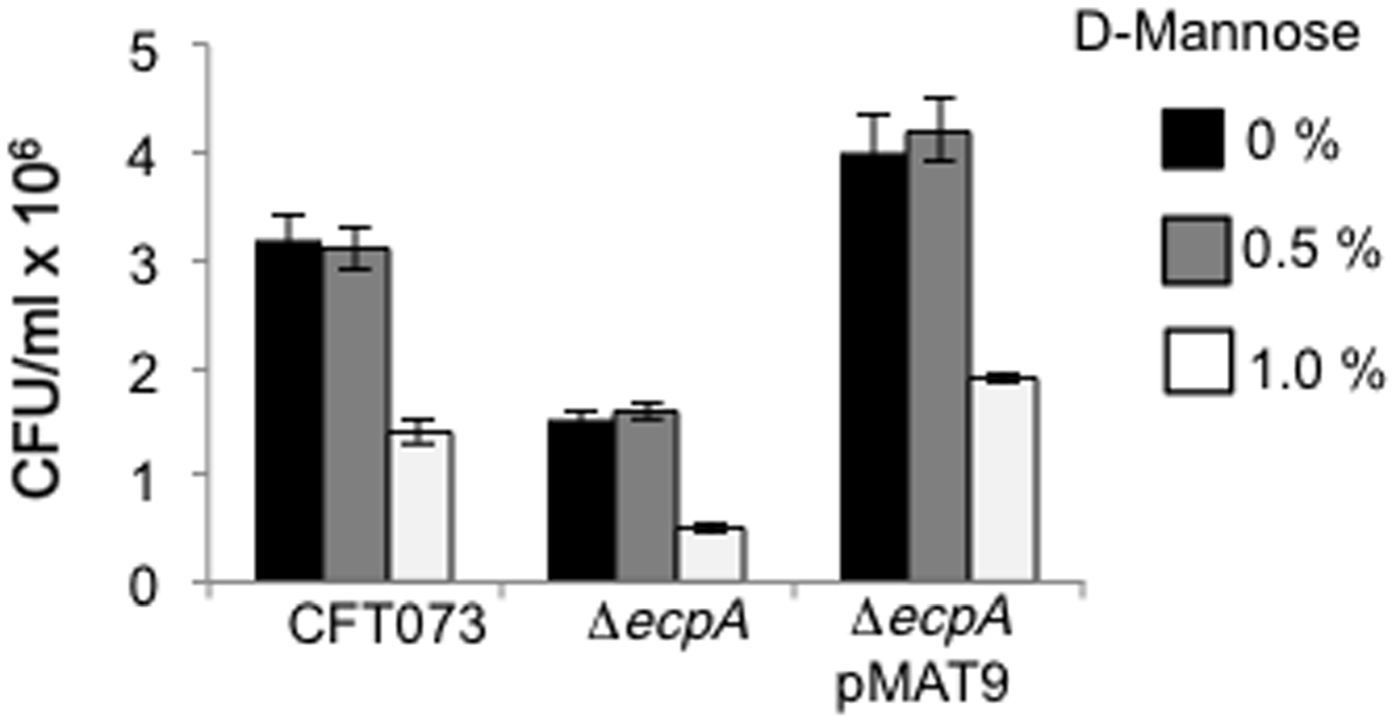
Effect of D-mannose on type 1 pili-mediated adherence. Adherence of CFT073 and derivative strains to HeLa cells cultured in DMEM at 37°C was quantitated in the presence of 0, 0.5, and 1% D-mannose. Colony-forming-units (CFU) were obtained after plating out ten-fold dilutions of 1% TritonX-100-treated cells. Standard deviations are represented by error bars and represent the average of all the results obtained from the 3 experiments performed.

T1P have been reportedly associated with the pathogenesis of UTI by UPEC strains and thus we were interested in knowing the contribution of these pili to the adherence of CFT073. To do this, we performed an adherence assay in the presence of 0, 0.5 and 1% D-mannose, which is known to inhibit T1P-mediated adherence. Adherence of CFT073, the *ecpA* mutant and the complemented strain, was only reduced significantly when 1% D-mannose was used indicating that T1P is responsible for about two thirds of the *ecpA* mutant residual adherence and that both T1P and ECP contribute to the adhesiveness of CFT073 (Fig. 5). We also performed yeast agglutination assays with WT, *ecpA* mutant and the complemented strain to compare levels of T1P among these backgrounds. T1P-mediated yeast agglutination was reduced similarly in the three strains in the presence of 0.5 and 1% D-mannose. The inhibition was higher with 1% D-mannose (data not shown). Since 1% D-mannose inhibited cell adherence and yeast agglutination we conclude that T1P levels produced are not affected in any of the strains either in the presence of epithelial cells or yeasts.

### ECP promotes invasion of murine bladder urothelium cells

**Figure 6:**
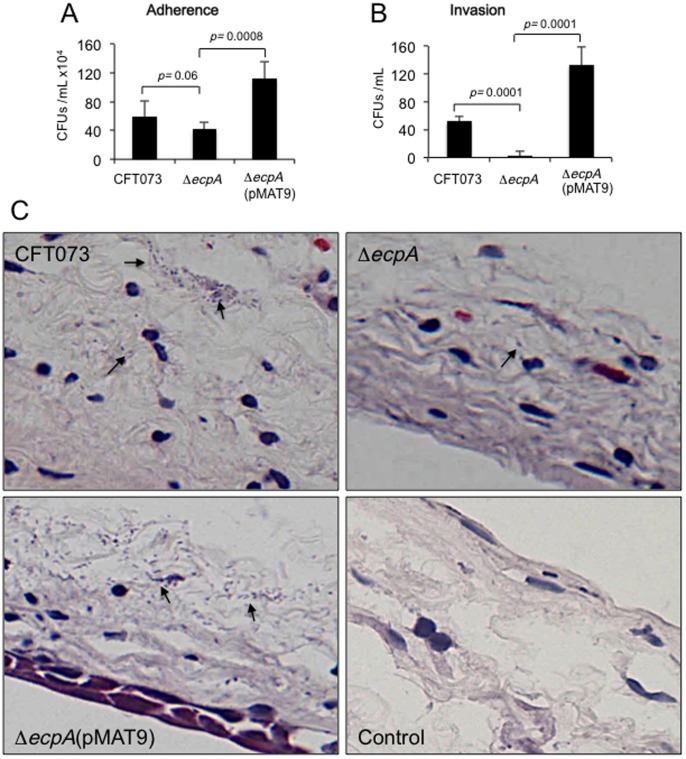
Adherence and invasion of UPEC CFT073 to mouse bladder urothelium tissue. (A) Quantitative comparison of adherence of CFT073, CFT073 Δ*ecpA*, CFT073 Δ*ecpA* (pMAT9) in DMEM at 37°C. (B) The gentamicin-protection (Invasion) assay was done to quantify the ability of UPEC strains to invade bladder urothelium from infant mice *ex vivo*. The difference in invasion between CFT073 vs. CFT073 Δ*ecpA* is considered to be statistically significant (*p*<0.0001). For panels A and B standard deviations are represented by error bars and represent the average of all the results obtained from the 3 experiments performed. (C) Histological sections of mice bladder urothelium infected *ex vivo* with bacteria of CFT073 wild-type, CFT073 Δ*ecpA* and CFT073 Δ*ecpA* (pMAT9) stained with Hematoxylin-Eosin. The arrows point to the bacteria present. Few to none bacteria were seen in the mutant and mock-infected tissues while numerous wild-type and complemented strain bacteria can be seen throughout the tissue. Images were taken with a 60× objective.

We set out to investigate whether the *in vitro* observations with cultured epithelial cells regarding production of ECP and adherence could be reproduced when infecting mice bladder urothelium *ex vivo*. In addition, we were interested in assessing and comparing the invasive capabilities of the CFT073 wild-type strain versus the *ecpA* mutant. To this purpose, we also used the gentamycin protection assay and sectioned the infected bladder tissues to visualize the bacteria and ECP production. We found that the CFT073 *ecpA* mutant was slightly deficient in adherence compared to the wild-type strain but did not reach significance (Fig. 6A). However, invasion of bladder epithelial cells by the *ecpA* mutant was strongly reduced (*p* = 0.0001), providing compelling evidence for the first time that ECP mediates invasion of UPEC (Fig. 6B). The microscopic analysis of the thin sections of bladder tissue infected with the wild-type and the complemented strains showed bacteria present along the edges of the tissue (extracellular) and also inside the tissue. The number of *ecpA* mutant bacteria was much less with respect to the wild-type strain or the complemented strain, while the complemented strain showed more bacteria than the parental and mutant strains (Fig. 6C). These qualitative observations correlate with the quantitative data shown in Fig. 6A and B.

### Evidence of ECP production in exfoliated bladder epithelial cells from UTI

**Figure 7:**
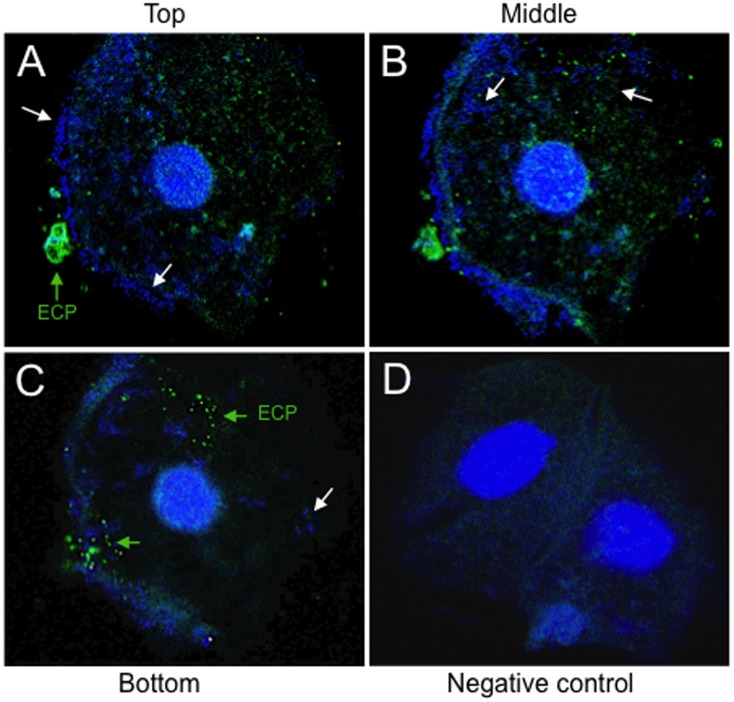
Confocal microscopy of an exfoliated bladder epithelial cell obtained from the midstream urine of a patient with UTI. ECP production was detected by confocal microscopy in urine samples of women clinically diagnosed with acute UTI. (A, B, C) The presence of ECP (green arrows, Alexa-Fluor 488) on the bacteria (white arrows, bacteria stained blue with DAPI) present on exfoliated epithelial cells was demonstrated using an anti-EcpA antibody (1:1,500). Note the presence of extra- and intracellular bacteria in the different layers (A, top; B, middle; C, bottom) obtained by confocal microscopy. (D) An urine sample from a clinically healthy woman was used as a negative control. Images were taken with a 40× objective of Zeiss LMS-510 laser scanning microscope.

We were interested in knowing if ECP are produced during natural UTI. Exfoliated bladder epithelial cells were obtained from the sediment of urine of an UTI patient and the presence of *E. coli* and ECP were manifested by IFM and confocal microscopy. Different layers of epithelial cells were analyzed. The micrographs show extra- and intracellular bacteria present at different layers of the cell and that some of the bacteria (single or in clusters) produce ECP (Fig. 7A–C). An urine sample from a clinically healthy woman was used as negative control and showed no bacteria or ECP (Fig. 7D). These data suggest that ECP may be produced *in vivo* in the bladder epithelium during the course of an UTI.

### Biofilms

**Figure 8:**
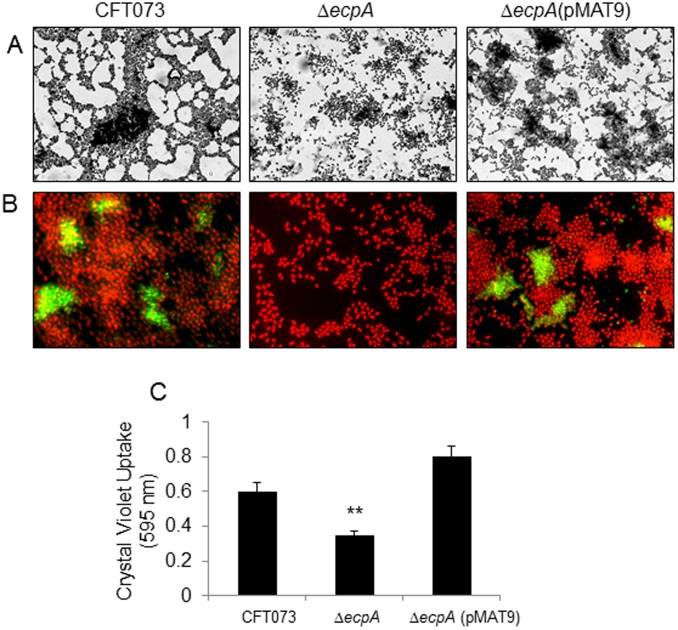
Role of ECP in biofilm formation. (A) Giemsa staining of biofilms of CFT073 and derivative strains showing a difference in the architecture of the biofilms formed after incubation of the bacteria for 24 h in DMEM at 37°C on glass coverslips contained in 24-well plates. (B) Immunofluorescence microscopy showing the presence of ECP in certain areas of the biofilms formed by CFT073 and CFT073 Δ*ecpA* (pMAT9). Images were taken with a 60× objective. (C) Quantification of biofilm formation by measuring Crystal Violet uptake. Standard deviations are represented by error bars and represent the average of all the results obtained from the 3 experiments performed. **, Statistically significant with respect to the wild-type strain (*p*<0.01).

Expression of pili, flagella, antigen 43, and polysaccharides has been reported to contribute to biofilm formation in *E. coli* and many other bacterial pathogens [31], [32], [33]. Since biofilm formation is a property of UPEC strains not only *in vitro* but also *in vivo* in the urinary tract [9], we were interested in knowing whether ECP was associated with the ability of UPEC to form biofilms on abiotic surfaces. When the CFT073 Δ*ecpA* was compared to its parental strain, we observed a clear difference in the pattern and architecture of the biofilms formed by the two strains (Fig. 8A). However, IFM studies showed that only a subset of bacteria within the biofilms of CFT073 and CFT073 Δ*ecpA* (pMAT9) displayed fluorescent pili (Fig. 8B). Quantification of biofilms supported the microscopical findings, such that the mutant was somewhat affected in biofilm formation as compared to the wild-type and the complemented strains (Fig. 8C). These data indicate that the production of ECP within the bacterial communities contributes to some degree to the shape and architecture of the biofilm and that other surface determinants are obviously involved in this phenomenon.

## Discussion

The interaction of UPEC with the host urinary tract is a multi-factorial and complex phenomenon that involves several distinct fimbrial adhesins that act in concert during different stages of infection [4], [5]. How these different adhesins are synchronized and differentially regulated within the context of space and time during the interplay of UPEC with host uroepithelial cells remains to be defined.

In this study, we show that the newly described ECP is yet another key player in the general scheme of the interaction of UPEC with host cells. The basis for this assumption comes from compelling IEM, IFM, and molecular data indicating that UPEC produces ECP when incubated with cultured epithelial cells and during biofilm formation on a glass surface. Most interestingly, ECP production was manifested with specific anti-ECP antibodies in the mouse bladder urothelium *ex vivo* and in exfoliated bladder epithelial cells present in the urine of an UTI patient, strongly suggesting that ECP genes could be expressed in the human urinary tract environment. The role of ECP in UPEC adherence was investigated by the phenotypic analysis of genetically defined *ecpA* mutants of two prototypic UPEC strains revealing a significant reduction in adherence to HeLa and HTB-4 cells. We could see only a partial reduction in adherence in the CFT073 and F11 *ecpA* mutants, which is likely attributed to the presence of redundant adhesins such as T1P and P pili in these strains. In fact, adherence assays performed in the presence of 1% (and not 0.5%) D-mannose significantly reduced T1P-mediated adherence of the wild-type and derivative isogenic strains. Thus, the data indicate that ECP works in synchronicity with other adhesins presumably to ensure host cell colonization.

Our data provokingly suggest that production of ECP may be necessary for stabilizing the extracellular bacteria adhering to the host cell membrane favoring tissue colonization and perhaps in intracellular biofilms. Other studies have shown that ECP is important for bacteria-to-bacteria interactions during biofilm formation of other *E. coli* pathogroups [21], [34]. Thus, it is likely that ECP are important for specific interactions between bacteria and for maintenance of the architecture of the bacterial community. The IFM images showing ECP expression on UPEC adhering to cultured epithelial cells and on intracellular *E. coli* in the mouse bladder urothelium and exfoliated bladder epithelial cells present in urine sediments from an UTI patient are intriguing and highly evocative of a role for ECP in the biology of this organism within the urinary tract of the host. The *ecpA* mutant had a drastic defect in invasion of HeLa and HTB-4 cells and the murine uroepithelium. There are numerous fibrillar structures that are associated with intracellular development in UPEC [9]. Integrin, CD14, and TLR were identified as the cellular molecules mediating T1P-mediated invasion [5].

*E. coli* has the ability to colonize many different niches within a host. This is mainly possible due to an assortment of cooperative and some times seemingly redundant adhesive structures. In this context, UPEC ECP may act only as an auxiliary adhesin at a particular stage of the infection in the human urinary tract, thus it remains to be determined whether ECP contributes to human UTI. Since a vast majority of the UPEC bacteria exit the urinary tract and then the host through urination after escape from the intracellular residence, it is possible also that ECP contributes to binding in the next environment that UPEC encounters after leaving the host. UPEC strains are commensal organisms in the gastrointestinal tract. The reported contribution of ECP in the binding of EHEC O157:H7 and fecal *E. coli* to intestinal epithelial cells *in vitro*, reasonably suggests that ECP could also be important for UPEC to establish a niche in the gut. The presence of ECP in the majority of *E. coli* strains [15] suggests that ECP function may not be specific only for human gastrointestinal disease, but it may also be important for survival in various environmental niches. We presented here compelling evidence that ECP is produced in the intracellular environment in the urothelium of mice (*ex vivo*) and in exfoliated cells present in the urine of UTI patients. The observation that ECP is common to most *E. coli* strains, including normal flora as well as human and pathogenic *E. coli* strains, suggests that it may not have a specialized role in any particular disease state, but contributes to the normal lifestyle that all *E. coli* strains share. Although intracellular residence is not a common lifestyle amongst all *E. coli* strains, living in a host certainly is.

In summary, this study shows that UPEC strains are capable of producing ECP under biologically relevant conditions and highlights a role for ECP as an accessory adherence factor, which in conjunction with other adhesins, may contribute to the multi-factorial complex interaction of UPEC with host uroepithelial cells.
